# Multiple responses optimization of antioxidative components extracted from Fenugreek seeds using response surface methodology to identify their chemical compositions

**DOI:** 10.1002/fsn3.2949

**Published:** 2022-06-07

**Authors:** Jie Yang, Zhenzhen Zhang, Qimeng Wu, Xiaoyuan Ding, Chenyang Yin, Endong Yang, Dongdong Sun, Weiyun Wang, Yunqiu Yang, Feng Guo

**Affiliations:** ^1^ School of Life Sciences Anhui Agricultural University Hefei China; ^2^ State Key Laboratory of Tea Plant Biology and Utilization Anhui Agricultural University Hefei China

**Keywords:** antioxidant activity, Fenugreek seeds, flavonoids, LC–MS, multiple response surface optimization, natural products

## Abstract

Fenugreek seeds (*Trigonella foenum‐graecum* L.), one kind of traditional Chinese medicine, are reported to be of great potential as a new alternative in terms of their bioactive components. In our present study, an ultrasonic‐assisted method was applied in the extraction of antioxidative components from fenugreek seeds. Four factors: ethanol concentration, liquid–solid ratio, sonication time, and sonication power were selected and multiple responses were studied using the response surface methodology (RSM). The effects of factors along with the correlation between all responses (flavonoids content, 2,2‐diphenyl‐1‐picrylhydrazyl (DPPH) assay, OH^−^ assay) were studied. The regression model indicated that all four factors are of significant effect on all responses. The model predicted that the ethanol concentration of 72%, solvent‐to‐material ratio of 35 ml/g, ultrasonic time of 41 min, and 500 W of power would provide a flavonoid yield of 9.10 mg/g, DPPH clearance of 80.33%, and OH^−^ clearance of 24.28%, respectively. The confirmation test showed the closeness of the predicted results with those of experimental values. And AB‐8 resin was successfully used to purify the fenuellus hulusi seed extract, and the flavonoid concentration of 78.14% was obtained. Six flavonoids (Swertisin, Puerarin apioside, Jasminoside B, Astragalin, Apigenin‐7‐O‐beta‐D‐glucoside, and Apiin) were successfully identified by the liquid chromatography–mass spectrometry (LC–MS) analysis.

## Graphical abstract

The extraction method of antioxidant components from Fenugreek seeds was optimized.The extract was purified by macroporous resin and the flavonoids were analyzed by LC‐MS.

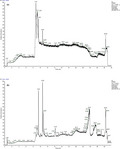

## 1 INTRODUCTION

Flavonoids are secondary metabolites that are ubiquitous in plants. Modern research shows that flavonoids are a common natural antioxidant. Their unique chemical structure makes them easy to be oxidized, thus showing strong in vivo antioxidant capacity. Studies have confirmed that the flavonoid Flavokawain B can protect cells from hydrogen peroxide (H_2_O_2_) damage by neutralizing reactive oxygen species (ROS) in the cells (Yeap et al., 2017). The study of Martin et al. found that 8‐prenylnaringenin has a strong antioxidant activity, showing the ability to protect colonic epithelial cells from oxidative stress damage (Ambrož et al., 2019). Upadhyay et al. found that 5‐O‐demethylnobiletin obtained from Okinawa plant extracts had good 2,2‐diphenyl‐1‐picrylhydrazyl (DPPH) free radical scavenging ability (Upadhyay et al., 2014), and the scavenging ability of the flavonoids to DPPH free radical showed a significant positive correlation with the concentration of the compound. Due to the various biological activities of flavonoids, they have received extensive attention in the field of health in recent years. Research has confirmed that free radicals are closely related to human cardiovascular disease, physiological aging, and other factors that threaten public health. They have a strong oxidizing property, which can cause oxidative stress in people and endanger their health (Furukawa et al., 2004; Mohammad‐Sadeghipour et al., 2020). Butylated hydroxyanisole (BHA) and butylated hydroxytoluene (BHT) are currently the most common synthetic antioxidants (Topal et al., 2015; Zhang et al., 2015). They are often used as food additives to prevent or delay food oxidation, improve food stability, and prolong the shelf life. However, toxicological experiments have shown that these synthetic antioxidants have relatively large toxic and side effects (Girgih, 2014), and have adverse effects on the human liver, spleen, and lungs. And antioxidant experiments also show that these commonly used synthetic antioxidants have weaker antioxidant effects than natural extracts. Side effects of synthetic antioxidants make it necessary to find a natural alternative.

Fenugreek seeds are internationally recognized as the most efficient edible and medicinal plant in the field of biological antioxidants (Kaviarasan et al., 2007). Modern pharmacological studies have shown that fenugreek seeds are a potential natural antioxidant plant (Madhava Naidu et al., 2011). Total flavonoids from fenugreek seeds can scavenge hydroxyl radicals (OH‐) and inhibit H_2_O_2_‐induced mitochondrial lipid peroxidation in rat hepatocytes. Trigonelline can inhibit the formation of tumor necrosis factor alpha (TNF‐α), cause hemoglobin glycation and lipid accumulation, and downregulate the gene expression of nicotinamide adenine dinucleotide phosphate (NADPH) oxidase and mitochondrial electron transport system (Tharaheswari et al., 2014; Prabhjot et al., 2021), and inhibit or slow down the oxidative stress of the body. In addition, fenugreek seed extract also has lipid, hypoglycemic, hypolipidemic, and cardiovascular disease related links with its antioxidant capacity (Omri et al., 2019; Sur et al., 2010; Khole et al., 2014; Reddy & Srinivasan, 2009). The flavonoids in fenugreek seeds, in particular, are the main source of fenugreek's antioxidant power. Therefore, the total flavonoids of fenugreek seeds have a high research value in an antioxidant.

Considering the high antioxidative potential of fenugreek seeds, extraction optimization of bioactive components is our priority. Recently, response surface methodology (RSM) has been widely used in the optimization of separation and extraction (Siewe et al., 2021; Briones‐Labarca et al., 2019; Zhou et al., 2018; Chen et al., 2018). Herein, a multiple responses surface methodology (MRSM) was applied to optimize the extraction of fenugreek seed. The effects of factors along with the correlation between all responses (flavonoids content, DPPH assay, OH^−^ assay) were studied.

## 2 MATERIALS AND METHODS

### 2.1 Materials

Fenugreek seeds were obtained from Anhui Dechang Pharmaceutical Co., Ltd. Dried seeds were ground into powder and then screened with a 60‐mesh sieve. Fenugreek seed powder was extracted by a Soxhlet extractor with petroleum ether (60–90°C) as the extractant, and after 30 min, petroleum ether was filtered out to obtain fenugreek seed powder. The powder was placed in a plastic bag and stored in the refrigerator at 4°C before use. All other chemicals and solvents (analytical and high‐performance liquid chromatography [HPLC] grades) were purchased from Shanghai Sinopharm Group Co., Ltd.

### 2.2 Ultrasound‐assisted extraction (UAE)

An ultrasonic processor (500 Watts and 40 kHz model, Yongkang Ultrasonic Co., Ltd.) was used. The extraction was performed using ethanol/water as a solvent in different proportions with a total extraction volume of 100 ml. After each extraction, the extracts were vacuum filtered on Whatman filter paper No. 4, and the filtrates stored at −18°C ± 2°C until analysis.

### 2.3 Experimental design

As in the ultrasonic‐assisted extraction (UAE) of fenugreek seeds, ethanol concentration (*X*_1_), solvent‐to‐material ratio (*X*_2_), ultrasonic time (*X*_3_), and ultrasonic power (*X*_4_) were selected as four main factors. Based on single‐factor experiment results, varying *X*_1_ (15%, 30%, 45%, 60%, 75%, and 90%, w/w), *X*_2_ (10, 15, 20, 25, 30, and 35 ml/g), *X*_3_ (5, 15, 25, 35, 45, and 55 min), *X*_4_ (0, 100, 200, 300, 400, and 500 W), and TFC (total flavonoids content) as quota, RSM was followed to optimize the extraction process using Design‐Expert Ver. 8.1.5 (Stat‐Ease Inc.). The Box–Behnken design (BBD) was applied to obtain the optimal values for the four main independent variables at three different levels (−1, 0, 1). Total flavonoids content, DPPH assay, and OH^−^ assay were considered as the response of the design experiments. Regression analysis was performed accurately based on the experimental data. Later, three additional confirmation extraction experiments under the optimal conditions were carried out to verify the accuracy of the statistical experimental strategies.

### 2.4 Total flavonoids content (TFC)

The total flavonoid in fenugreek seed extract was measured according to the technique described by the sodium nitrite–sodium nitrate aluminum hydroxide colorimetric method. Rutin was used to give the standard curve: *y* = 12.54*x*−0.0104 and *R* ^2^ = .9979, where *y* is the absorbance value of the sample and *x* is the sample concentration. TFC was expressed as the rutin equivalent (RE) per gram of FPFS (freeze‐drying powders of Fenugreek seeds) (milligrams (mg) RE/g) and calculated by Equation (1).

(1) $$\mathrm{TFC}=\frac{\text{The flavonoids content of extracts}\;\left(\mathrm{mg}\;\mathrm{RE}\right)}{\text{weight of FPFS}\;\left(g\right)}$$

### 2.5 Antioxidant activities

All antioxidative effects were determined by using techniques which have already been reported.

The DPPH clearance rate could be calculated as follows:

(2) $$\text{scavenging percentage}\%=\frac{A_{0}-\left(A_{1}-A_{2}\right)}{A_{0}}\times 100$$

2,2‐diphenyl‐1‐picrylhydrazyl (0.05 mg/ml): 0.1 mg of DPPH was dissolved into 20 ml of absolute ethanol solution. *A*_0_: A mixture solution of anhydrous ethanol and DPPH, *A*_1_: A mixture solution of sample and DPPH, *A*_2_: A mixture solution of sample and anhydrous ethanol.

The OH removal rate could be calculated as follows:

(3) $$\text{scavenging percentage}\%=\frac{A_{0}-\left(A_{1}-A_{2}\right)}{A_{0}}\times 100$$

A: 6 mmol ethanol‐salicylic acid, 7.5 mmol ferrous sulfate solution, and 4% H_2_O_2_ solution were mixed at a ratio of 1:1:1. A_0_: A mixture solution of A and distilled water. A_1_: A mixture solution of A and sample. A_2_: A mixture solution of distilled water and sample.

### 2.6 Identification of antioxidant substances in extracts

After optimizing the extraction method, the Fenugreek seeds were extracted under the optimal conditions, and the extraction solution was configured as 0.5 mg/ml, and the pH was adjusted to 5. An AB‐8 macroporous resin was used to adsorb the extract, and 3 bV (bed volumes) of 40% ethanol was used for elution after the adsorption. The eluent was collected step by step in 5 ml units, and flavonoid content in the eluent was determined successively. The eluent with a higher flavonoid content was combined and analyzed by LC–MS (Samoticha et al., 2017).

Thermo Scientific Hypersil Gold (100 mm × 2.1 mm, 1.9 μm) was selected as the chromatographic column, and gradient elution was carried out with 0.075% formic acid water (A) and acetonitrile (B) as mobile phase. The gradient changes were 10%–12% B 0–10 min, 12%–16% B 10–20 min, 16%–25% B 20–25 min, 25%–40% B 25–35 min, 40%–78% B 35–45 min, and 78%–10% B 45–50 min, 10% B 50–55 min. Flow rate was 0.2 ml /min, the column temperature was 40°C, and the injection volume was 2 μl. Electrospray ionization (ESI): full MS‐DDMS2 acquisition mode was adopted for mass spectrometry. Ion transfer tube temperature was 350°C; spray voltage of 3.4 KV (+)/3.0 KV (−); sheath gas 45 arb; auxiliary gas 15 arb; a 200–1500 mass/charge m/z ratio range; resolution of 70,000; and MS2 fragmentation voltages of 20, 30, and 60 eV.

## 3 RESULTS AND DISCUSSION

### 3.1 Single‐factor experiments

**FIGURE 1:**
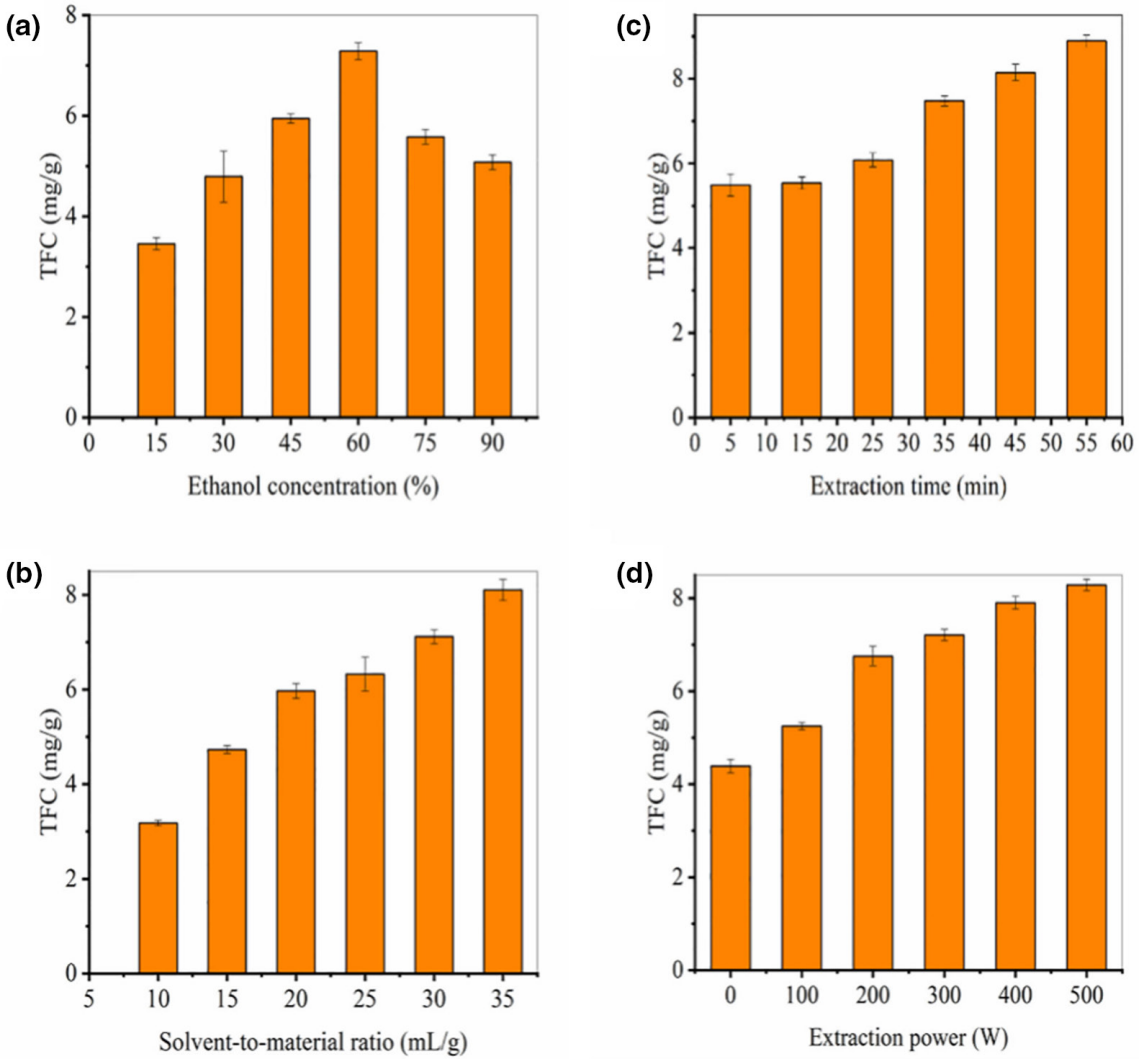
Effects of ethanol concentration (a), solvent‐to‐material ratio (b), ultrasonic time (c), and ultrasonic power (d) on the total flavonoid concentration (TFC). Results were expressed as average values ± standard deviation (*n* = 3)

Flavonoids were commonly considered as an important natural antioxidant in different herbs. Thus, the total flavonoids content (TFC) was selected as a quota in the present single‐factor experiments. Influences of different ethanol/water systems, solvent‐to‐material ratio, ultrasonic time, and ultrasonic power were investigated. Results indicated that the extraction efficiency was affected by ethanol concentration, ultrasonic time, power, and solvent‐to‐material ratio (Figure 1).

When the ethanol concentration increased from 15% to 60%, the TFC increased significantly, and after more than 60%, the extraction rate began to decline, indicating that the extraction rate of total flavonoids from fenugreek seeds was the highest at this concentration (Figure 1a); solvent‐to‐material ratio is one of the factors affecting TFC (Figure 1b). When the liquid‐to‐solid ratio increases from 10 ml/g to 20 ml/g, TFC increases rapidly. At 25 ml/g, TFC grows slowly, and when the liquid‐to‐material ratio exceeds 25 ml/g, its concentration tends to increase, so the contact area of solvent and solid reaches saturation with the increase of solvent‐to‐material ratio; the ultrasonic time had a significant effect on the extraction of TFC (Figure 1c). When the ultrasonic time increased from 5 min to 25 min, the TFC did not increase significantly, while the TFC increased rapidly at 25–55 min, especially at 25–55 min. The fastest growth occurred at 35 min. One possible explanation is that the flavonoids can be released rapidly within 25–35 min and accumulated rapidly in the extract, therefore, the ultrasonic time of at least 35 min is more suitable for the extraction of total flavonoids from fenugreek seeds; the increase of ultrasonic power had a strong effect on the increase of TFC (Figure 1d). When the ultrasonic power was increased from 200 W to 500 W, the extraction rate of TFC in fenugreek seeds increased gradually from 0 W to 200 W, with the increase of power, the mechanical force and thermal effect caused by cavitation during the extraction process resulted in the rupture of the cell wall of fenugreek seeds and accelerated the transfer of flavonoids contained in the cells to the solvent. This finding may explain the total amount of fenugreek seeds. That is the reason for the high extraction rate of flavonoids.

### 3.2 Optimizing extraction conditions by the Box–Behnken design

Response surface methodology (RSM) was performed to optimize the extraction conditions using Design‐Expert Ver. 8.1.5 (Stat‐Ease Inc.). The Box–Behnken design (BBD) was used to find the optimal values for four independent variables: ethanol concentration, ultrasonic time, power, and solvent‐to‐material ratio at three different levels (−1, 0, +1) (Table 1).

**TABLE 1:** Factors and levels in response surface methodology (RSM)

Independent variable	Symbol	Level		
		−1	0	1
Ethanol concentration (%)	*X*_1_	35	62.5	90
Solvent‐to‐material ratio (ml/g)	*X*_2_	15	22.5	35
Ultrasonic time (min)	*X*_3_	15	35	55
Ultrasonic power (W)	*X*_4_	100	300	500

The interactions between these four main factors could be obtained by this experimental design. The entire study comprised 29 separate experiments and each treatment was tested in triplicate.

As shown in Table 2, the yield of TFC from fenugreek seeds was 2.3 (90%, 10 ml/g, 35 min, 300 W) –10.394 mg RE/g (62.5%, 35 ml/g, 55 min, 300 W) with rutin as the quantitative standard. The results were similar to the yield (1.03%) obtained by Yang Renming et al. (Baba et al., 2018). However, both DPPH clearance rate (66.58%–89.35%) and the yield of flavonoids were significantly higher than the 6.76 mg quercetin equivalent (QE)/g dry weight (DW) (59.9%) reported by Qazi Tabasum et al., and the results obtained by Norziah, M. H et al. (68%) using the Soxhlet extraction method (Norziah & Fezea, 2015).

**TABLE 2:** Designed experiments and measured responses of the response surface analysis

Run	*X*_1_ (%)	*X*_2_ (ml/g)	*X*_3_ (min)	*X*_4_ (W)	Y_TFC_ (mg/g)	Y_DPPH_ (%)	Y_OH_ (%)
1	90	22.5	15	300	3.919	76.77	5.27
2	62.5	22.5	35	300	5.48	81.68	21.19
3	62.5	35	35	500	8.189	82.14	28.06
4	62.5	22.5	35	300	6.269	80.88	20.65
5	90	22.5	35	500	3.99	76.09	10.61
6	35	22.5	35	100	4.959	69.44	44.91
7	62.5	10	35	500	3.534	82.17	17.57
8	35	22.5	15	300	5.946	89.35	15.08
9	62.5	35	15	300	6.542	88.11	18.93
10	90	35	35	300	6.375	79.16	7.58
11	90	22.5	35	100	2.358	74.98	4.86
12	62.5	10	35	100	3.967	81.89	12.82
13	62.5	22.5	35	300	5.049	78.96	14.2
14	62.5	22.5	55	500	7.346	79.14	31.94
15	90	22.5	55	300	4.941	75.75	3.55
16	62.5	22.5	35	300	5.856	78.5	14.21
17	62.5	22.5	15	500	4.169	82.99	17.38
18	35	10	35	300	3.177	85.07	16.42
19	62.5	22.5	15	100	4.144	83.11	19.47
20	35	35	35	300	8.022	76.29	27.88
21	62.5	22.5	55	100	4.356	74.9	15.81
22	62.5	35	55	300	10.394	67.71	20.3
23	62.5	35	35	100	6.734	76.48	31.21
24	62.5	10	55	300	3.911	83.22	0.73
25	62.5	10	15	300	4.635	79.95	4.05
26	90	10	35	300	2.3	80.5	6.77
27	62.5	22.5	35	300	5.856	78.05	14.02
28	35	22.5	55	300	5.785	66.58	32.88
29	35	22.5	35	500	4.762	86.62	26.71

The regression coefficients of the quadratic predicted model are indicated in Table 3.

**TABLE 3:** Regression coefficient (β) and fit statistics of the predicted second‐order polynomial models for flavonoids and antioxidant activity

Factor	Coefficient (β)		
	TFC	DPPH	OH^−^
*F* value (model)	25.83^***^	9.84^***^	7.44^***^
*F* value (lack of fit)	1.19	2.64	2.19
Intercept	5.70	79.61	16.85
*X*_1_	−0.73^***^	−0.84	−10.44^***^
*X*_2_	2.06^***^	−1.91^**^	6.30^***^
*X*_3_	0.61^***^	−4.42^***^	2.09
*X*_4_	0.46^**^	2.36^**^	0.27
*X*_1_ *X*_2_	−0.19	1.86	−2.66
*X*_1_ *X*_3_	0.30	5.44^***^	−4.88
*X*_1_ *X*_4_	0.46^*^	−4.02^**^	5.99^*^
*X*_2_ *X*_3_	1.14^***^	−5.92^***^	1.17
*X*_2_ *X*_4_	0.47	1.34	−1.98
*X*_3_ *X*_4_	0.74^**^	1.09	4.56
*X*_1_^2^	−0.97^***^	−1.83	−0.64
*X*_2_^2^	0.44^*^	1.43	−1.92
*X*_3_^2^	0.22	−0.46	−2.79
*X*_4_^2^	−0.72^**^	−0.16	6.71^**^
*R* ^2^	.9627	.9078	.8816
Adj. *R* ^2^	.9255	.8155	.7631
*p*‐value (lack of fit)	.4701	.1815	.2344
*p*‐value (model)	<.0001	<.0001	.0003

*Note: X*_1_: ethanol concentration (%); *X*_2_: solvent‐to‐material ratio (ml/g); *X*_3_: ultrasonic time (min); *X*_4_: ultrasonic power (W).

Abbreviations: TFC, total flavonoids content; OH^−^, hydroxyl radical scavenging ability by the salicylic acid method; DPPH, 2‐2‐dipheny‐1‐picrylhydrazyl radical scavenging capacity.

^*^ *p* < .05: indicates significant level.

^**^ *p* < .01: indicates highly significant level.

^***^ *p* < .001: indicates remarkably significant level.

The quadratic polynomial equation was fitted by response surface regression as follows:

(4) $$Y=\beta_{0}+\sum_{i=1}^{k}\beta_{i}X_{i}+\sum_{i=1}^{k}\beta_{\mathrm{ii}}X_{\mathrm{ii}}^{2}+\sum_{i}^{k-1}\sum_{j}^{k}\beta_{\mathrm{ij}}X_{i}X_{j}$$

where Y is the response variable; $X_{i}\;$ and $X_{j}$ are independent variables; $\beta_{0}$ is the constant coefficient; $\beta_{i}\;$ is the linear coefficient; $\beta_{\mathrm{ii}}$ is the quadratic coefficient; and $\beta_{\mathrm{ij}}$ is the cross‐product coefficient.

The **DPPH‐RSC** (radical scavenging activity) is mainly related to *X*_2_ *X*_3_, followed by *X*_2_ *X*_3_ *X*_1_ *X*_3_, *X*_3_, *X*_1_ *X*_4_, *X*_4_, *X*_2_. Table 3 shows that the *F* value of model Y_DPPH‐RSC_ is 9.84 with a *p*‐value <.0001, indicating that the model is significant and can be used for subsequent optimization designs. Moreover, the *p*‐values of *X*_1_ *X*_4_, *X*_4_, *X*_2_ are lower than .01; thus, these factors significantly impact Y_DPPH‐RSC_. The *p*‐values of *X*_2_ *X*_3_ *X*_1_ *X*_3_, *X*_3_ are lower than .001, showing that the influence of ethanol concentration and ultrasonic time on DPPH‐RSC is exceptionally significant. **OH‐RSC** is mainly related to *X*_1_, *X*_2_, followed by *X*_4_^2^, *X*_1_ *X*_4_. Table 3 shows that the model Y_OH‐RSC_ has an *F*‐value of model Y_OH‐RSC_ of 7.44, with a *p*‐value <.0001, indicating that the model is significant and can be used for subsequent optimization designs. These results also mean that *X*_4_^2^, *X*_1_ *X*_4_ (*p*‐value <.05) have a significant impact on OH‐RSC. Specifically, the linear effect of *X*_1_, *X*_2_ shows to be highly significant (*p*‐value <.0001). Table 3 shows that the *F* value of model Y_TFC_ is 25.83 with a *p*‐value <.0001, indicating that the model is significant and can be used for subsequent optimization designs. Also, *X*_2_^2^, *X*_4_ values (*p*‐value <.05) indicated a major influence on TFC. More specifically, the linear effect of ethanol concentration, ultrasonic time, and solvent‐to‐material ratio (*X*_1_, *X*_2_, *X*_3_) implied a remarkably significant (*p* < .001) positive effect on TFC.

The analysis of variance (ANOVA) for the experimental outcomes of the BBD was also presented distinctly (Table 3). A low *p*‐value (*p* < .0001) indicated that this predicted model was statistically significant. The *R* ^2^ is useful for checking the model fitness, being in this study classified as strong (*R* ^2^ adjust was relatively close to 1), except for OH^−^ that showed a *R* ^2^ adjusted lower than .80. The *R* ^2^ values were, respectively, 96.27%, 90.78%, and 88.16% on the TFC yield, DPPH and OH^−^ clearance rate responses. The *p*‐values for the lack‐of‐fit model were nonsignificant (*p* > .05), which also supported our assumption that the predicted model was sufficient to accurately represent the experimental data.

Based on the multiple regression analysis of all experimental data, a second‐order polynomial equation was performed to express the predicted model. Equations (5)–(7), respectively, show the dependence of TFC yield, DPPH and OH^−^ clearance rates on ethanol concentration (*X*_1_), solvent‐to‐material ratio (*X*_2_), ultrasonic time (*X*_3_), and ultrasonic power (*X*_4_). The parameters of the regression model equation were determined through/for the responses and variables in accordance with the multiple regression analysis of the experimental results.

(5) $$\text{YTFC}=5.70-0.73X_{1}+2.06X_{2}+0.61X_{3}+0.46X_{4}-0.19X_{1}X_{2}+0.30X_{1}X_{3}+0.46X_{1}X_{4}+1.14X_{2}X_{3}+0.47X_{2}X_{4}+0.74X_{3}X_{4}-0.97X_{1}^{2}+0.44X_{2}^{2}+0.22X_{3}^{2}-0.72X_{4}^{2}$$

(6) $$\text{YDPPH}=79.61-0.84X_{1}-1.91X_{2}-4.42X_{3}+2.36X_{4}+1.86X_{1}X_{2}+5.44X_{1}X_{3}-4.02X_{1}X_{4}-5.92X_{2}X_{3}+1.34X_{2}X_{4}+1.09X_{3}X_{4}-1.83X_{1}^{2}+1.43X_{2}^{2}-0.46X_{3}^{2}-0.16X_{4}^{2}$$

(7) $$\mathrm{YOH}=16.85-10.44X_{1}+6.30X_{2}+2.09X_{3}+0.27X_{4}-2.66X_{1}X_{2}-4.88X_{1}X_{3}+5.99X_{1}X_{4}+1.17X_{2}X_{3}-1.98X_{2}X_{4}+4.56X_{3}X_{4}-0.64X_{1}^{2}-1.92X_{2}^{2}-2.79X_{3}^{2}+6.71X_{4}^{2}$$

### 3.3 Analysis of the response surface

**FIGURE 2:**
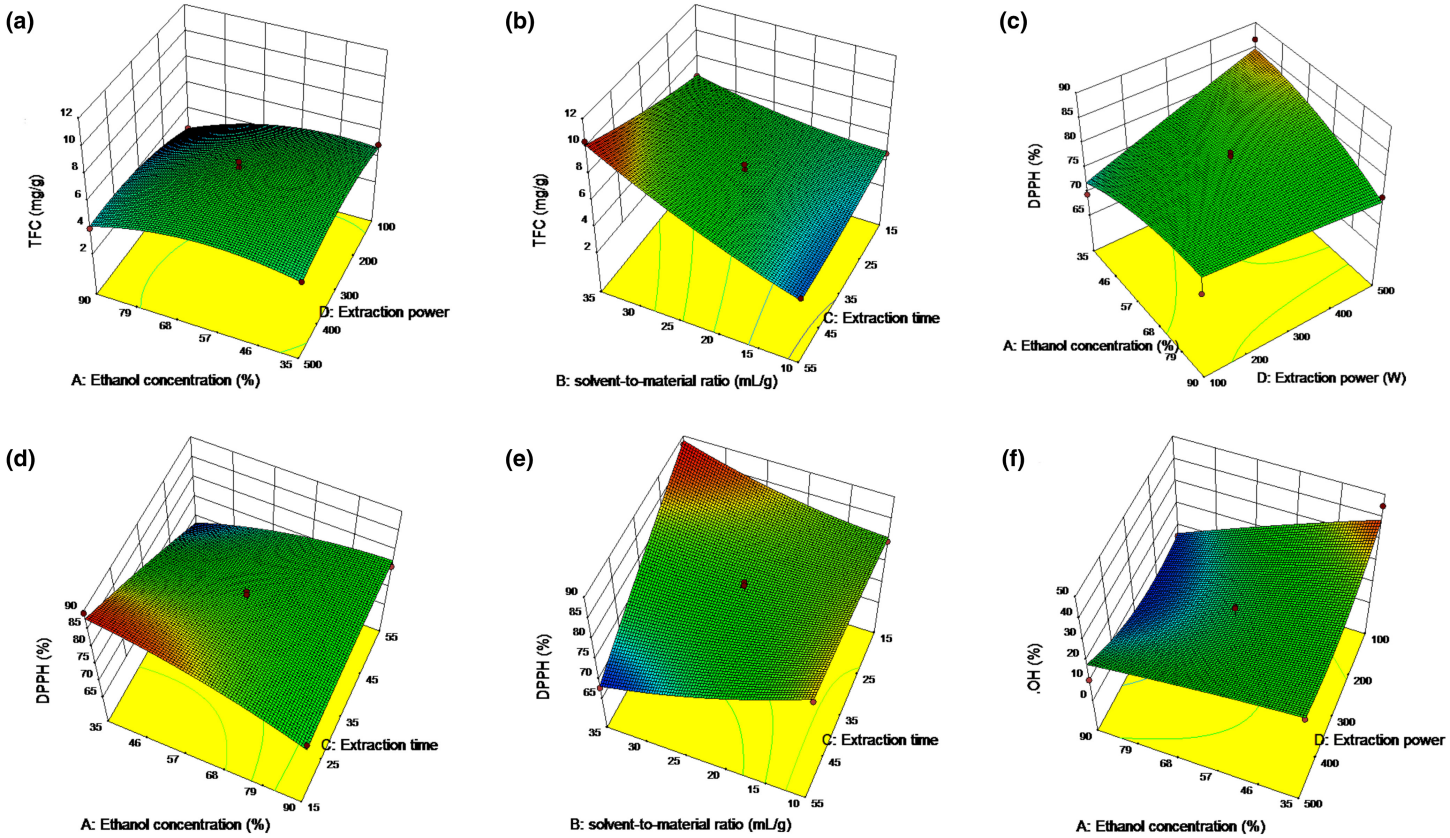
Response surface three‐dimensional (3D) plots for the interaction effects of independent variables of ethanol concentration (%), solvent‐to‐material ratio (ml/g), extraction ultrasonic time (min), and ultrasonic power (W) on dependent variables of total flavonoids content (TFC) (a, b), 2‐2‐dipheny‐1‐picrylhydrazyl (DPPH) (c–e), and OH^−^ (f)

It can be seen from Figure 2a that the interaction of *X*_1_ and *X*_4_ has a significant positive effect on TFC, and the interaction of ethanol concentration and ultrasonic power (*X*_1_ *X*_4_) has a significant (*p* < .05) positive effect on TFC. TFC was gradually increased at lower ethanol concentration and lower ultrasonic power. However, with the increase of ultrasonic power and ethanol concentration, the increase of TFC gradually flattened and started to decrease, which may be because TFC is a polar compound and is more soluble in solvents with similar polarities (Chen et al., 2018). As the concentration increases and decreases, the polarity of the extract also decreases and increases, thereby reducing the TFC. With the increase of ultrasonic power, the cell walls of fenugreek seeds are more easily broken, and it is easier to release flavonoids (He et al., 2016). When the ultrasonic power is too high, the compounds in the fenugreek will accumulate more bonds, resulting in the destruction of the compound structure. Therefore, with the continuous increase of ultrasonic power, TFC will show a downward trend. The solvent‐to‐material ratio and extraction time showed a significant negative effect on TFC. It can be seen from Figure 2b that with the increase of the solvent‐to‐material ratio, the TFC showed a continuous upward trend, and the increase of TFC was in the case of a longer extraction time and it will be more pronounced. This shows that increasing the liquid–solid ratio during extraction is very important to improve the TFC yield, because with the increase of the solvent amount, the diffusion pressure also increases, which promotes as many flavonoid molecules as possible to dissociate into the solution, and the extraction rate is correspondingly increased (Yan et al., 2011). With the improvement of cavitation, the particle collisions generated by cavitation can also destroy the cell wall better and promote the exudation of components (Zhang et al., 2019). In the case of a longer extraction time, flavonoids are also more likely to be precipitated, which makes the cavitation effect more obvious.

As can be seen in Figure 2c, the interaction of *X*_1_ *X*_4_ has a significant effect on DPPH‐RSC, which is consistent with TFC. And the three‐dimensional (3D) map also appears to be consistent with Figure 2a, which indicates that the DPPH free radical scavenging ability in fenugreek seed extract mainly comes from the flavonoids, and the scavenging ability is positively correlated with the content of flavonoids. Figure 2d shows that the interaction of ethanol content and extraction time has a significant effect on the free radical scavenging ability of DPPH. When the ethanol content was low, DPPH‐RSC continued to decrease with the increase of extraction time, and when the ethanol content was higher, DPPH‐RSC continued to increase with the increase of extraction time. This may be because when the ethanol concentration is low, the large polar molecules in the extract are more easily precipitated, and with the extension of the extraction time, the proportion of flavonoids in the extract decreases, which shows a decrease in antioxidant capacity. When the ethanol concentration increased, the flavonoids with a similar polarity were more easily dissolved. The interaction of *X*_2_ *X*_3_ (Figure 2e) also showed a significant effect on DPPH‐RSC, which validates the results of Figure 2b. However, TFC showed differences with DPPH‐RSC, which may be due to the rapid release and accumulation of flavonoids in the extract due to the increased solvent–material contact area and prolonged sonication time. However, with the increase of liquid–material ratio and ultrasonic time, the antioxidant activity decreased, and with the increase of ultrasonic time, the antioxidant activity decreased rapidly. The reason may be that the extraction time is too long, which leads to a change in the structure of flavonoids and the dissolution of other impurities, which not only reduces the content of total flavonoids, but also reduces the antioxidant activity.

Ethanol concentration meter extraction power also showed a significant interaction in the hydroxyl radical scavenging rate (Figure 2f), consistent with Figure 2a. Under the condition of ethanol concentration of 90% and ultrasonic power of 100 W, the response values of TFC content and OH‐scavenging rate were significant. Possibly due to the increase of *X*_1_ and the decrease of polarity, some flavonoids with a higher polarity could not be dissolved, and the yield of TFC and the scavenging rate of OH‐radicals decreased. Under the same conditions, the DPPH clearance rate was increased, and the flavonoids with lower polarity may have a better effect on the DPPH clearance rate (Norlia et al., 2014; Yang et al., 2002). The interaction of ethanol concentration and sonication time had a significant effect on the DPPH clearance, which indirectly confirmed this speculation.

### 3.4 Identification of compounds

**FIGURE 3:**
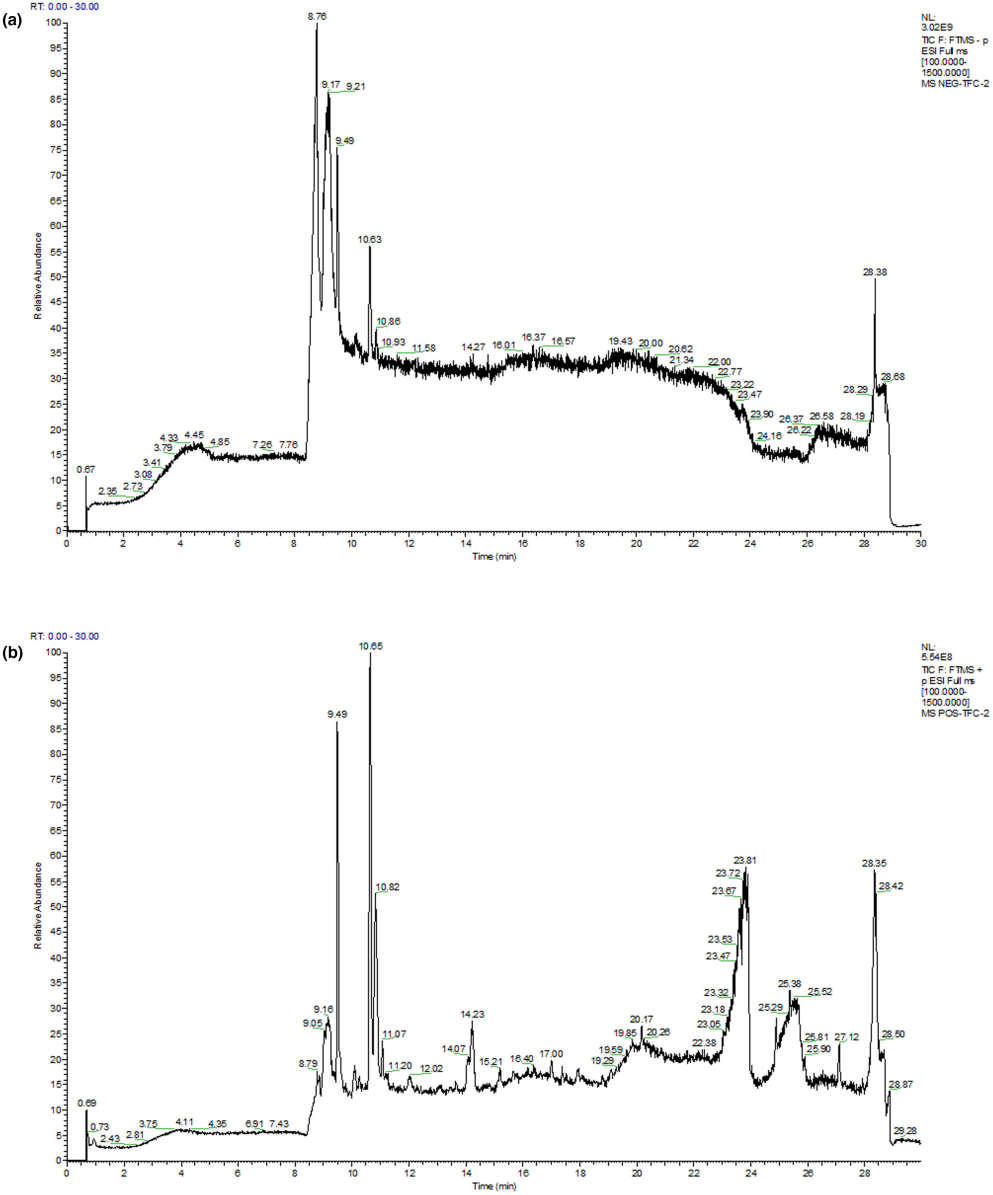
Negative ion flow (a) and positive ion flow patterns (b)

The extract of Fenugreek seeds was purified by AB‐8 resin and the flavonoid content of 71.84% was obtained. Six flavonoids, such as Swertisin, Puerarin apioside, Jasminoside B, Astragalin, Apigenin‐7‐O‐beta‐D‐glucoside, and Apiin, were successfully identified by the LC–MS analysis. The related information of each compound is listed in Table 4, and the total ion current spectrum is shown in Figure 3.

**TABLE 4:** Chemical compositions of extracts from Fenugreek seeds

RT(min)	Molecular weight	Formula	Compound name	Area (max.)
10.63	358.12	C_22_ H_22_ O_10_	Swertisin	13357.75
9.03	548.15	C_26_ H_28_ O_13_	Puerarin apioside	20061.67
14.07	346.16	C_16_ H_26_ O_8_	Jasminoside B	3736786.11
8.88	448.09	C_21_ H_20_ O_11_	Astragalin	92886.13
9.01	433.10	C_21_ H_20_ O_10_	Apigenin‐7‐O‐beta‐D‐glucoside	1205700.90
8.84	564.14	C_26_ H_28_ O_14_	Apiin	7067379.42

### 3.5 Model validation

A MRSM method was used to determine the optimal combination of each factor and level. The optimum extraction conditions were determined by Design‐expert software. The optimal conditions for extracting TFC and its antioxidant activity were as follows: ethanol concentration of 72%, solvent‐to‐material ratio of 35 ml/g, ultrasonic time of 41 min, and ultrasonic power of 500 W.

According to the improved optimal process conditions, verification experiments were repeated three times; the results are shown in Table 5. The experimental values and predicted values were basically consistent, indicating that the model parameters obtained by BBD optimization were accurate and reliable.

**TABLE 5:** Experimental values and predicted values of response variables at optimum extraction conditions

Response variables	Optimum extraction conditions	Experimental value	Predicted value
	*X*_1_ *X*_2_ *X*_3_ *X*_4_		
Y_TFC_	72% 35 ml/g 41 min 500 W	9.10 ± 0.13	8.89
Y_DPPH_		80.33 ± 0.41	79.27
Y_OH_		24.28 ± 0.22	25.19

*Note: X*_1_: ethanol concentration (%); *X*_2_: solvent‐to‐material ratio (ml/g); *X*_3_: ultrasonic time (min); *X*_4_: ultrasonic power (W). The experimental results were all expressed as mean ± standard deviation (*n* = 3).

## 4 CONCLUSION

Herein, an extraction method was applied to optimize the extraction of fenugreek seeds. The effects of factors along with the correlation between all responses (flavonoids content, DPPH assay, and OH^−^ assay) were studied.

The medicinal value of total flavonoids in fenugreek seed obtained by ultrasound‐assisted multiple responses surface methodology (MRSM) technology and their use as natural antioxidants were assessed in this study. According to a multiple response surface, the optimal extraction parameters of TFC yield and antioxidant activity of fenugreek under different conditions were determined, and the parameters were *X*_1_ = 72%, *X*_2_ = 35 ml/g, *X*_3_ = 41 min, and *X*_4_ = 500 W. Under these optimal conditions, the extraction flavonoid yield of 9.10 mg/g, DPPH clearance of 80.33%, and OH^−^ clearance of 24.28%, respectively, were observed. In addition, the results showed that the ultrasonic‐assisted multiresponse surface methodology was used to optimize the extraction of TFC yield and antioxidant activity was significantly improved. Flavonoids are thought to be a natural antioxidant that protects against many diseases caused by the oxidative stress. Overall, these results provide new insights into the extraction of flavonoids from fenugreek seeds and the study of antioxidant activity. And AB‐8 resin was successfully used to purify fenuellus hulusi seed extract, and the flavonoid concentration of 78.14% was obtained. Six flavonoids (Swertisin, Puerarin apioside, Jasminoside B, Astragalin, Apigenin‐7‐O‐beta‐D‐glucoside, and Apiin) were successfully identified by the LC–MS analysis.

## CONFLICT OF INTEREST

The authors declare that there are no conflicts in interest.

## ETHICAL APPROVAL

Ethical approval is not applicable for this article.

## STATEMENT OF HUMAN AND ANIMAL RIGHTS

This article does not contain any studies with human or animal subjects.

## STATEMENT OF INFORMED CONSENT

There are no human subjects in this article and informed consent is not applicable.
